# 288. Prognostic Factors for In-hospital Mortality of Patients with Severe COVID-19 Hospitalized in the University Medical Center, Lithuania

**DOI:** 10.1093/ofid/ofac492.366

**Published:** 2022-12-15

**Authors:** Ieva Kubiliute, Jurgita Urboniene, Akvile Rudenaite, Birute Zablockiene, Giedre Gefenaite, Ligita Jancoriene

**Affiliations:** Clinic of Infectious Diseases and Dermatovenerology, Institute of Clinical Medicine, Faculty of Medicine, Vilnius University, Vilnius, Lithuania, Vilnius, Vilniaus Apskritis, Lithuania; Center of Infectious Diseases, Vilnius University Hospital Santaros Klinikos, Vilnius, Lithuania, Vilnius, Vilniaus Apskritis, Lithuania; Center of Infectious Diseases, Vilnius University Hospital Santaros Klinikos, Vilnius, Lithuania, Vilnius, Vilniaus Apskritis, Lithuania; Clinic of Infectious Diseases and Dermatovenerology, Institute of Clinical Medicine, Faculty of Medicine, Vilnius University, Vilnius, Lithuania, Vilnius, Vilniaus Apskritis, Lithuania; Department of Health Sciences, Faculty of Medicine, Lund University, Lund, Sweden, Lund, Skane Lan, Sweden; Clinic of Infectious Diseases and Dermatovenerology, Institute of Clinical Medicine, Faculty of Medicine, Vilnius University, Vilnius, Lithuania, Vilnius, Vilniaus Apskritis, Lithuania

## Abstract

**Background:**

Till May 2022 there were 1.06 million of COVID-19 cases and 9108 deaths caused by COVID-19 disease registered in Lithuania. The objective of the study was to evaluate prognostic factors for in-hospital mortality of patients with severe COVID-19 disease.

**Methods:**

COVID-19 positive adult patients hospitalized in Vilnius University Hospital Santaros Klinikos, Lithuania, were included in the cohort study between March 2020 and December 2021. Medical history, vital signs were collected, laboratory tests, chest X-ray were performed. Severe COVID-19 disease was defined as pneumonia with objective respiratory failure symptoms. ROC curve was used to evaluate prognostic value of a laboratory test, multivariable logistic regression was used to determine the prognostic factors for in-hospital lethal outcome. p-value < 0.05 was considered significant.

**Results:**

Severe COVID-19 disease was diagnosed for 291 participants, 21 (7.2%) of them died. Hematological, lung diseases and cancer were more common among patients who died compared to those who recovered (Table 1). Patients who died were older, more frequently had confusion (25.0% *vs* 5.2%, p=0.001), tachypnea (57.1% *vs* 26.7%, p=0.003), and dyspnea (81.0% vs 54.1%, p=0.017), their time median from symptoms onset to admission was shorter (2.5 (IQR 0-7) *vs* 7 (IQR 4-9) days, p< 0.001) compared those who recovered. On admission, patients who died had lower lymphocytes count, higher neutrophiles count and higher concentration of aspartate aminotransferase (AST), lactate, creatinine and urea compared to those who recovered. The best prognostic features for lethal outcome possessed neutrophiles count, AST, lactate, creatinine and urea (Figure 1). Optimal cut-off values were calculated and included into multivariable regression model. Multivariable regression revealed that hematological diseases (OR 8.68; 95% CI 1.46-51.40, p=0.017), initial AST > 32.9 U/l (OR 13.57; 95% CI 1.46-125.94, p=0.022), lactate > 1.46 mmol/l (OR 4.87; 95% CI 1.03-22.86, p=0.045) were associated with in-hospital mortality of patients with severe COVID-19 adjusted for age.

**Figure:**
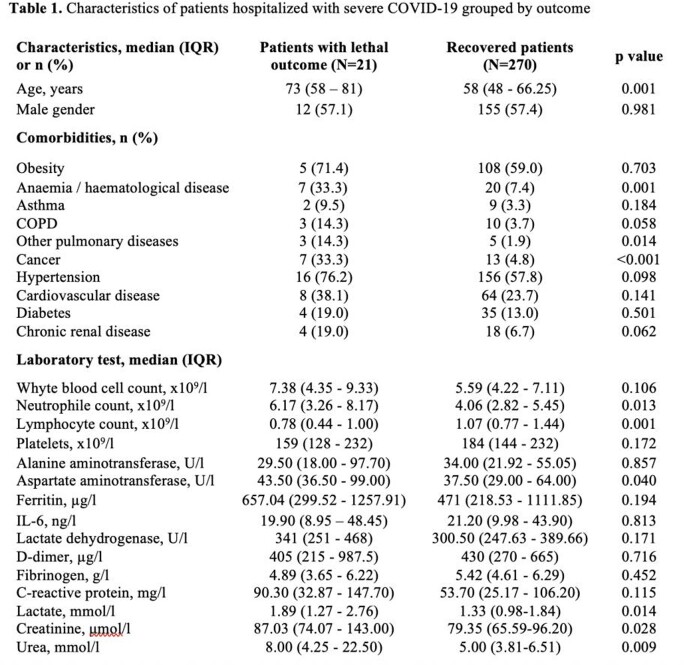

**Figure:**
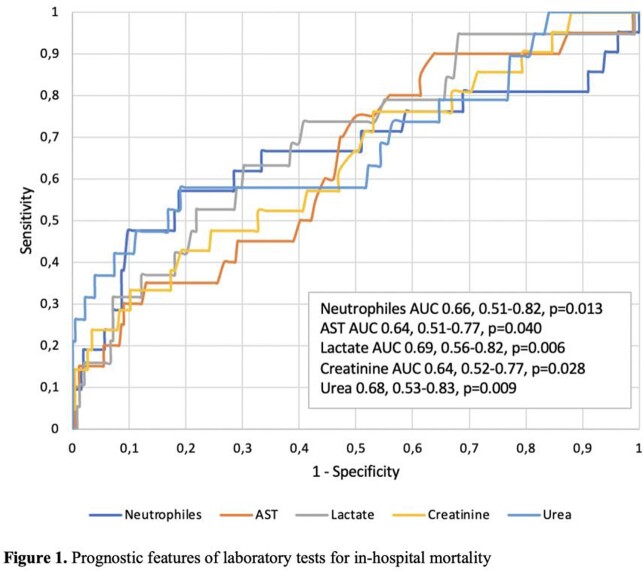

**Conclusion:**

Hematological diseases, elevated AST and lactate are significant prognostic factors for in-hospital mortality of patients with severe COVID-19 disease at earlier stages of disease.

**Disclosures:**

**All Authors**: No reported disclosures.

